# Upper Limb Kinematics Using Inertial and Magnetic Sensors: Comparison of Sensor-to-Segment Calibrations

**DOI:** 10.3390/s150818813

**Published:** 2015-07-31

**Authors:** Brice Bouvier, Sonia Duprey, Laurent Claudon, Raphaël Dumas, Adriana Savescu

**Affiliations:** 1 Institut National de Recherche et de Sécurité (INRS), 54519 Vandoeuvre-lès-Nancy, France; E-Mails: laurent.claudon@inrs.fr (L.C.); adriana.savescu@inrs.fr (A.S.); 2 Université de Lyon, F-69622 Lyon, France; E-Mails: sonia.duprey@univ-lyon1.fr (S.D.); raphael.dumas@ifsttar.fr (R.D.); 3 Université Claude Bernard Lyon 1, Villeurbanne, France; 4 IFSTTAR, UMR_T9406, LBMC Laboratoire de Biomécanique et Mécanique des Chocs, F69675 Bron, France

**Keywords:** sensor-to-segment calibration, ambulatory system, joint angle, magneto-inertial measurement unit sensors, accuracy

## Abstract

Magneto-Inertial Measurement Unit sensors (MIMU) display high potential for the quantitative evaluation of upper limb kinematics, as they allow monitoring ambulatory measurements. The sensor-to-segment calibration step, consisting of establishing the relation between MIMU sensors and human segments, plays an important role in the global accuracy of joint angles. The aim of this study was to compare sensor-to-segment calibrations for the MIMU-based estimation of wrist, elbow, and shoulder joint angles, by examining trueness (“close to the reference”) and precision (reproducibility) validity criteria. Ten subjects performed five sessions with three different operators. Three classes of calibrations were studied: segment axes equal to technical MIMU axes (TECH), segment axes generated during a static pose (STATIC), and those generated during functional movements (FUNCT). The calibrations were compared during the maximal uniaxial movements of each joint, plus an extra multi-joint movement. Generally, joint angles presented good trueness and very good precision in the range 5°–10°. Only small discrepancy between calibrations was highlighted, with the exception of a few cases. The very good overall accuracy (trueness and precision) of MIMU-based joint angle data seems to be more dependent on the level of rigor of the experimental procedure (operator training) than on the choice of calibration itself.

## 1. Introduction

Quantitative evaluation of upper limb kinematics is required in various applications such as rehabilitation, medicine, sport, and ergonomics. For such evaluations, several technological solutions associated with dedicated protocols of use are now proposed in the literature [1,2]. The optoelectronic system certainly represents the most widespread and mature non-invasive technology currently available, with many protocols having been proposed in the last two decades [3,4], including international standards (International Society of Biomechanics, ISB) [5]. Usually, this technology is based on infra-red cameras that track the 3D position of reflective markers placed on the subject. Although the high measurement accuracy of this technology is not challenged (0.1 mm in position, [6]), some limitations of use are involved such as lengthy camera calibration time, the restricted area of measurement (*i.e.*, within a calibrated space), and marker occlusions, often restricting its use to controlled laboratory environments [7].

It is thus necessary to turn to a more flexible device when monitoring ambulatory measurements is needed. In the past 10 years, much research has pointed to the potential use of (Magneto)-Inertial Measurement Unit sensors (MIMU) [8,9,10,11,12,13,14,15]. This technology is not subject to measurement area restriction and occlusion problems. Technically speaking, a MIMU sensor consists of a 3D accelerometer, a 3D gyroscope, and a 3D magnetometer. The accelerometer provides information on the linear accelerations of the MIMU sensor, including gravity. It can be used on its own for inclination measurement, but only under quasi-static conditions [16], and without providing information on movement around the vertical axis (called the Yaw axis or the Heading angle) [11]. The gyroscope is sensitive to angular rates but is subject to drift effects when the velocity is integrated to obtain angle information [17]. The magnetometer measures a magnetic field and can thus detect the magnetic north, which can be a source of information for the orientation around the vertical axis, assuming the absence of ferro-magnetic elements in the close environment [18,19]. Combining acceleration to angular velocity information considerably reduces the drift effect of gyroscopes. However, the orientation around the vertical axis remains inaccurate [11,19]. For a drift-free 3D orientation estimation, a third source of information must be added. Much research has highlighted the benefits of combining magnetometer to overcome the drawbacks of accelerometers and gyroscopes [18,19,20], although other alternative proposals exist such as the use of potentiometer [14], anatomical constraints [11], or global optimization process [21]. The concept underlying the functioning of the MIMU is the combination of these three types of information (accelerometers, gyroscopes, magnetometers) through sensor-fusion algorithms [20,22] to estimate a drift-free 3D orientation of the MIMU sensor. Geometrically, MIMU sensor 3D orientation is represented by a technical coordinate system (CS) expressed with respect to an earth-based global CS. This global CS is defined using the gravity (measured by the accelerometers) and the magnetic north (measured by the magnetometers) reference vectors [16] and thus it is common to all MIMUs. The relative motion between two consecutive segments can be calculated by attaching a MIMU sensor on each body segment of interest. However, this motion measurement has no anatomical signification and so cannot be converted into interpretable data such as joint angles (*i.e.*, flexion/extension, abduction/adduction, *etc.*) unless appropriate calibration is performed.

Thus, when focusing on joint kinematics, the definition of the segment CS must be associated with the MIMU CS [9]. International standards [5] do not provide a usable method due to the absence of position information delivered by MIMU. A calibration is therefore needed to establish the relation between each MIMU technical CS orientation and the corresponding human segment on which it is attached (segment CS). This “sensor-to-segment” calibration can be performed in three different methods. First, rigorous positioning of the sensor on a human segment, basically done by aligning the sensor edges in relation to the anatomy, allows assuming that the segment axes are equal to technical MIMU axes (TECH). This method is rarely used in the literature [8], or even mentioned as such. However, the advantage of TECH calibration is that it does not require any complementary computational step. Second, by asking the subject to maintain a specific static pose, e.g., standing with upper limbs along the body, all the segment axes, and so all the segment CS, can be generated as aligned all together (STATIC). STATIC calibrations are frequently used in the literature [8,9,23,24] since they are easy and quick to perform, consider an anatomical pose, and do not require strict MIMU sensor orientation on human segments. Third, another calibration method is to ask the subject to perform a series of specific functional movements during which segment axes can be estimated (FUNCT). The functional method, which, incidentally, represents an alternative to anatomical landmark-based ISB proposals [25], perfectly suits the use of the MIMU technology since only orientation is required (*i.e.*, no position). Several FUNCT calibrations for upper limb kinematics can be identified in the literature such as elbow flexion-extension, forearm pronation-supination, shoulder flexion-extension, and abduction-adduction [8,9,10].

Despite much interest in this topic from the scientific community, two aspects of research deserve more investigation regarding MIMU-based upper limb kinematics measurements. First, few studies have investigated the comparison of calibrations as they either focus on segment axes and not joint angles [9,15] or are performed on an artificial arm [10]. The work of De Vries *et al.* [9] certainly represents the most exhaustive comparative study. Based on a repeatability study performed on six subjects, they proposed a set of calibration steps for generating the segment axes for the thorax, upper arm, forearm, and hand. However, this study focused on segment axis definition rather than on final joint angle estimation and did not include the TECH calibration in the comparison. Second, performance evaluations of MIMU-based upper limb kinematics often appear incomplete and disparate in terms of the protocol and validity criteria used [7,8,11,26]. The protocols reported in the literature vary in terms of movement performed (*i.e.*, flexion-extension, abduction-adduction), amplitude of motion (submaximal, maximal or not mentioned), and the reference method (optoelectronic system, electromagnetic system) used for validation. Regarding the validity criteria used, validation of MIMU-based upper limb kinematics is generally restricted to the assessment of trueness (“close to a reference”), by using the non-invasive optoelectronic system as gold-standard. Referring MIMU-based data to those obtained by such a reference is certainly of interest to ensure communication between researchers. However, a systematic bias can be expected because of the different technologies and constructions used for the segment CS (position *vs.* orientation measurements as well as calibrations) [9]. Moreover, it is important to keep in mind that all non-invasive techniques suffer from soft tissue artefact (STA) effects, meaning that the reference data is not systematically representative of the underlying bone movements [27]. In addition, for a complete evaluation of MIMU-based upper limb kinematics, precision should also be considered a key criterion. By definition, high precision implies low data dispersion independently of how close the data are to the reference. This raises the question of repeatability/reproducibility. To the authors’ knowledge, only De Vries *et al.* [9] and Parel *et al.* [28] have investigated such a criterion for MIMU-based upper limb kinematics in adults, focusing on the repeatability of segment axes and on the reproducibility of the scapulohumeral rhythm, respectively.

As a general observation, it is difficult for the researcher/the practitioner (1) to know which sensor-to-segment calibration should be chosen from the literature as well as (2) to know what the global accuracy of MIMU-based upper limb kinematic data is.

The present study aims to answer these two points by proposing a comparative assessment of the sensor-to-segment calibrations for the MIMU-based estimation of wrist, elbow, and shoulder (humero-thoracic) joint angles. Calibrations were selected from the literature, mainly from the work of De Vries *et al.* [9], and include TECH, STATIC, and FUNCT classes of calibration. An exhaustive characterization of the accuracy of joint angles is approached by considering in the comparison both trueness (“close to a reference system”) and precision (reproducibility) validity criteria and by using an experimental protocol that includes subject and operator variability.

## 2. Methods

### 2.1. Participants

Ten healthy subjects (male, age 29 ± 3.4 years, height 180 ± 5 cm, weight 72 ± 6.7 kg), and three operators (A,B,C) participated in this study. The subjects had no history of right upper limb complaints and gave their informed consent for this experiment.

### 2.2. Technologies Used

A total of four wireless MIMU sensors (MTw, version firmware 2.0.8, Xsens, Enschede, Netherlands) were used for this experiment. The MTw is a small and light device (34.5 × 57.8 × 14.5 mm, 27 g) that consists of a 3D linear accelerometer, a 3D rate gyroscope, and a 3D magnetometer. Each MIMU communicates its data to a station (Awinda, Xsens) connected to a laptop, via a Bluetooth protocol communication. The MIMU technical CS with respect to an earth-based global CS is provided as an output, using Xsens fusion algorithm. Since MIMU data quality can be affected by magnetic disturbance, a homogeneous magnetic environment was maintained during the experiment. An optoelectronic system (Eagle 4, Motion Analysis Corporation, CA, USA) with 10 cameras was used as the reference system for measuring the kinematics (REF). Seventeen reflective markers were placed on bony landmarks, conforming to ISB standards [5]. The glenohumeral rotation center (GH) was estimated by regression [29]. Both systems were automatically synchronized using an external trigger-button at a sampling frequency of 60 Hz.

### 2.3. Experimental Protocol

**Figure 1:**
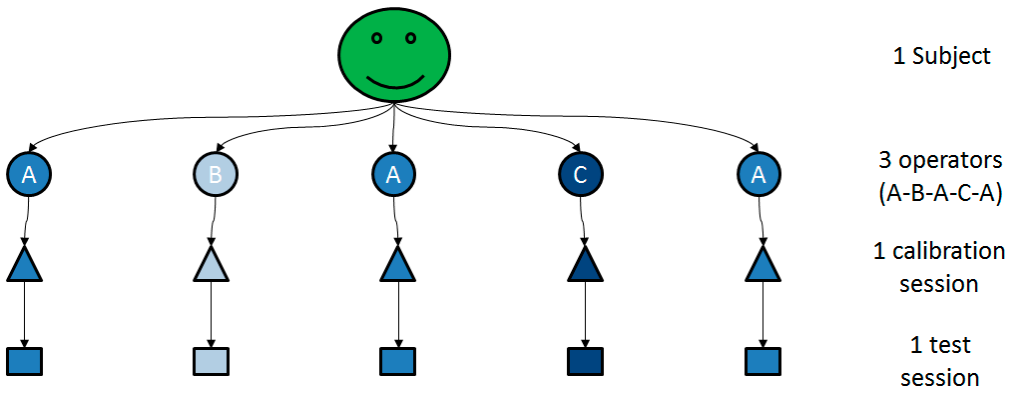
Experimental protocol for one subject.

At the beginning of the experiment, the subject was equipped with the reflective markers; they were not removed or replaced throughout the experiment. Then, he had to perform five experimental sessions, each composed of a calibration session and a test session. The five calibration sessions were supervised by three different operators (A,B,C) in the following fixed order A-B-A-C-A (Figure 1).

**Figure 2:**
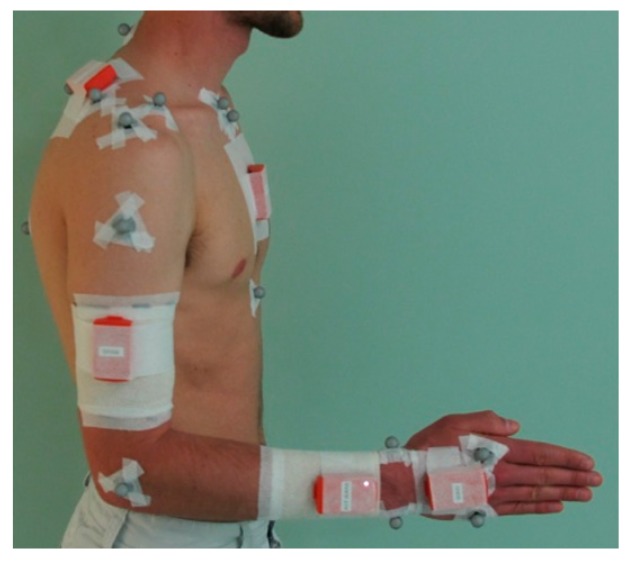
MIMU sensor placement and attachment on a subject.

A calibration session (triangle, Figure 1) consisted of two main steps. In the first step, the operator had to place and attach the four MIMU sensors on the subject: on the flat portion of the sternum [8], on the central third of the right upper arm [8], laterally (or slightly posterior if judged useful to reduce the occurrence of STA) [8], dorso-distally on the right forearm [8,9,11], and dorsally on the right hand [9] (Figure 2). MIMU sensors were attached using double-side tape and a sticky elastic band. Remark: the MIMU sensor placed on the scapula (see Figure 2) was not used for this study.

In the second step, the subject had to perform the seven following calibration steps, according to the instructions given by the operator:

IN A STANDING POSITION

- Pose STATIC_1: upper limbs along the body, in neutral forearm pronation-supination (PS), fingers pointing downwards;
- Pose STATIC_2: upper arm along the body, elbow flexed at 90°, in neutral forearm PS, fingers pointing forward;
- elbFE: starting from STATIC_2, performing right elbow flexion-extensions (FE), with a limited amplitude of [−30°~+30°] around 90°;
- elbPS: starting from STATIC_2, performing right forearm PS, with a complete amplitude, keeping an elbow FE of 90°, hand clenched in a fist.

IN A SEATED POSITION

- handF: maintaining the posture with right forearm and right hand flat on a table;
- wriE: starting from handF, performing right wrist extensions, with a limited amplitude of [0°~30°];
- shoIE: performing right shoulder internal/external rotations (IER), with a limited amplitude of [−10°~+30°], using a handle sliding on a table.

All postures were maintained for five seconds. All movements corresponded to series of five (launched) consecutive uni-axial rotations performed at moderate speed.

The operator was responsible for the smooth operation of the whole calibration session, using dedicated written instructions. If a calibration step was not judged in conformity by the operator, it was re-executed until reaching conformity.

**Figure 3:**
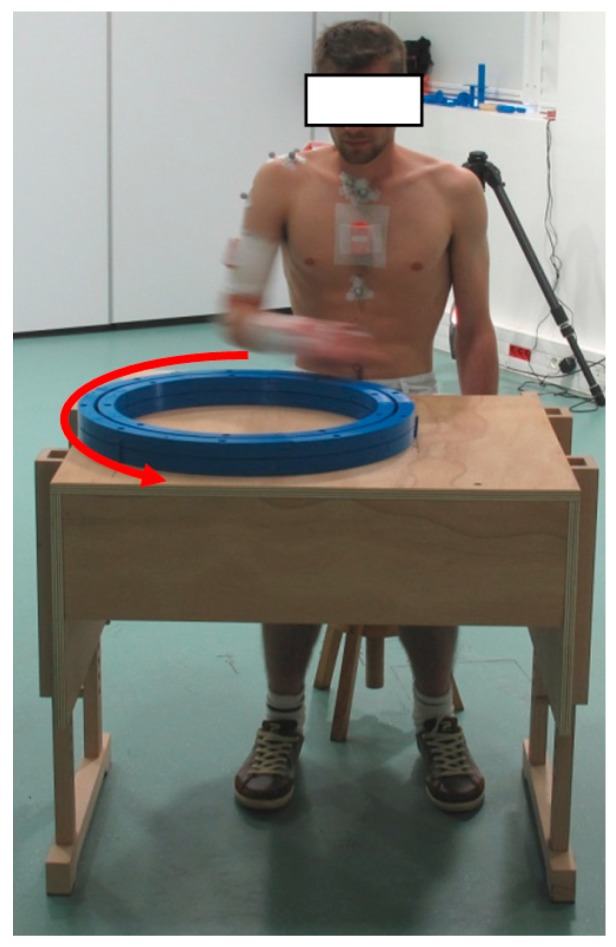
Illustration of the wheel movement.

Each calibration session was followed by a test session (rectangles, Figure 1). During the test session, the subject had to perform the six following test movements:

- wriFEmax: starting from STATIC_2, performing maximal wrist flexion-extensions;
- wriAAmax: starting from STATIC_2, performing maximal wrist abduction-adductions;
- elbFEmax: starting from STATIC_2, performing maximal elbow flexion-extensions;
- shoFmax: starting from STATIC_1, performing maximal shoulder flexions;
- shoAmax: starting from STATIC_1, performing maximal shoulder abductions in the scapular plane;
- wheel: seating, performing rotations using a circular wheel placed in front of him (Figure 3).

All the test movements were performed with the right limb only. Test movements of maximal amplitude corresponded to a series of 16 (launched) consecutive rotations, performed freely in the air at moderate speed. Regarding the shoulder abduction movement, the scapular plane was defined as 30° anterior to the frontal plane [30,31]. A dedicated piece of wood forming a 30° angle was used to indicate the plane of elevation expected to the subject just before he started the movement.

The wheel used in the experiment was a circle 50 cm in diameter, composed of a handle moving along a circular rail (Figure 3). The wheel movement corresponded to a series of 20 consecutive full anti-clockwise circles of the wheel performed in the horizontal plane. The subject was asked to perform this movement in a natural manner at moderate speed, keeping the handle in his right hand. The wheel was made of plastic and the table and the seat used were made of wood to exclude any possible ferro-magnetic disturbance.

### 2.4. Modeling

#### 2.4.1. Sensor-to-Segment Calibrations

The calibration session allowed for using different methods to establish the relation between the technical MIMU CS and the segment CS. For the sake of clarity, two classes of calibration were distinguished: calibrations used for defining all full segment CS (full-segment calibration) and calibrations dedicated to the definition of a single segment axis (one-axis calibration). Using a strict sensor placement, all segment CS can be considered equal to the technical MIMU CS (TECH). The two poses (STATIC_1 and STATIC_2) allowed us to generate all the segment CS by first defining the thorax CS, assuming the technical Z axis of the MIMU sensor placed on the thorax belonged to the sagittal plane, and secondly by duplicating this CS to all the other segments. Thus, TECH, STATIC_1 and STATIC_2 represented the three possible full-segment calibrations. Conversely, the elbFE, elbPS, wriE, and shoIE movements and handF pose were dedicated to the definition of a single axis, here called one-axis calibrations. The symmetrical axis of rotation approach (SARA) [32] and the gravity vector estimation were used for the axis definition during these movements and during this pose, respectively. Although elbFE, elbPS, wriE, and shoIE movements correspond to functional calibrations (FUNCT), these explicit denominations will be kept for the sake of clarity.

Calibrations were selected from the calibration session by using either a full-segment calibration (TECH, STATIC_1, STATIC_2) or a combination of a full-segment calibration and one-axis calibration(s) (e.g., STATIC_1+elbFE), depending on which final joint definition they were involved in (Table 1). In the latter case, the axis defined by the one-axis calibration replaced its corresponding axis in the full-segment calibration. This concept of one-axis calibration is consistent with the idea that the segment axes are considered independent if they are not enforced orthogonal. Thus non-orthonormal segment representation was adopted (see below). A total of nine, nine, and five calibrations were selected for the wrist, elbow, and shoulder joint, respectively (Table 1).

**Table 1:** The sensor-to-segment calibrations selected for each joint studied.

	WRIST	ELBOW	SHOULDER (Humero-Thoracic)
**TECH**	TECH	TECH	TECH
**STATIC**	STATIC_1	STATIC_1	STATIC_1
	STATIC_2	STATIC_2	STATIC_2
**FUNCT**	STATIC_1 + wriE	STATIC_1 + elbFE	STATIC_1 + shoIE
	STATIC_1 + handF	STATIC_1 + elbPS	STATIC_2 + shoIE
	STATIC_1 + wriE + handF	STATIC_1 + elbFE + elbPS	
	STATIC_2 + wriE	STATIC_2 + elbFE	
	STATIC_2 + handF	STATIC_2 + elbPS	
	STATIC_2 + wriE + handF	STATIC_2 + elbFE + elbPS	

#### 2.4.2. Definition of the Segment Coordinate Systems (SCS)

**Figure 4:**
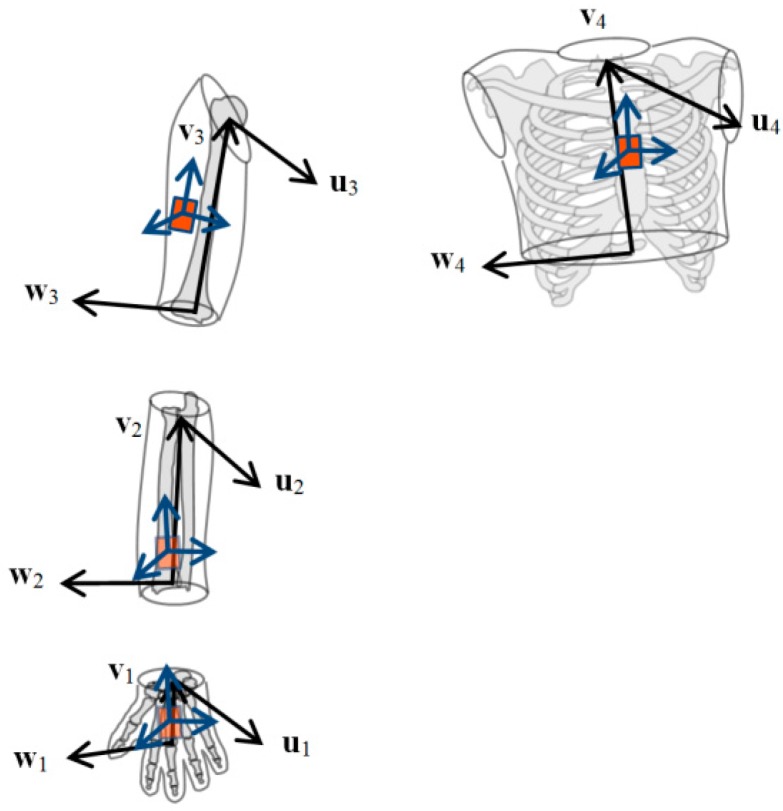
The non-orthonormal segment representation (*u*,*v*,*w*) used for the hand, forearm, upper arm, and thorax segments.

A common non-orthonormal segment representation was associated with all the MIMU sensor-to-segment calibrations as well as with the REF method. This representation consisted in defining a segment using three non-orthonormal axes: *u*, *v* and *w* [33]. Axis *u* is normal to the frontal plane of the segment, *v* is longitudinal (from the distal joint to the proximal joint), and *w* is called a flexion axis (*i.e.*, aligned with the functional axis when estimated or defined lateral, otherwise, pointing to the right) (Figure 4). Transformations from the non-orthonormal axes *u*, *v*, *w* to three orthonormal axes *x*, *y*, *z* allow defining different segment CS (with either *x* = *u*, *y* = *v*, or *z* = *w*) which is convenient in order to define the joint coordinate system according to different sequences of rotation [34].

#### 2.4.3. Definition of the Joint Coordinate Systems (JCS)

The Euler angle method, which consists in defining the relative motion of two adjacent segments in three consecutive rotations, was used. The first rotation was performed around a proximal axis (*z* = *w*), the third one around a distal axis (either *y* = *v* or *x* = *u*), and the second one around an axis perpendicular to these two axes, called a “floating” axis [5]. The *ZXY* sequence used for the elbow joint was that proposed by the ISB. For the wrist joint, the *ZYX* sequence was preferred in order to consider the internal-external rotation axis as the floating one (forearm pronation-supination was assimilated with the elbow internal-external rotation), as it has already been proposed for the ankle in the literature [35,36]. Concerning the humero-thoracic joint, here called “shoulder,” the choice of the Euler sequence proposed by the ISB is still under debate in the literature due to the considerable mobility of this joint, which can involve mathematical singularities [37]. Since motion around 0° and 180° elevation are of interest and all shoulder movements are composed of movements in the sagittal plane, the *ZXY* sequence was chosen [25].

### 2.5. Statistical Analysis

Joint angles were calculated for all the calibrations and the REF method during the six test movements (see test session) as well as during the calibrated movement of elbPS since it was performed with maximal amplitude. The trueness of each calibration, *i.e.*, difference with REF, and the precision of all the methods, including the REF, were assessed in this study.

#### 2.5.1. Assessment of Trueness

The trueness of the MIMU-based upper limb kinematics obtained using the different calibrations was assessed using two statistical indexes: the Coefficient of Multiple Correlation inter-protocol (CMCip) and the Root Mean Square Error (RMSE). The CMCip index, proposed by Ferrari *et al.* [38] based on the initial work of Kadaba *et al.* [39], quantifies the similarity between each MIMU-based joint angle waveform and the REF joint angle waveform, by taking into account differences in shape, offset, correlation, and range of motion [28,38]. Similarity in the waveforms results in the CMCip index tending towards “1”, whereas dissimilar waveforms result in the CMCip value tending towards “0”. Four mean CMCip thresholds were considered: very good (≥0.85), good (0.75–0.85), moderate (0.65–0.75), and bad (<0.65) [40]. Both indexes, CMCip and RMSE, were calculated for each session using the entire dataset and then averaged over the whole experiment (N = 50 sessions).

#### 2.5.2. Assessment of Precision

The precision of upper limb kinematics obtained using the MIMUs with the different sensor-to-segment calibrations and with the REF method was assessed using two approaches: the Coefficient of Multiple Correlation within-protocol (CMCwp) [38] and the approach proposed by Schwartz *et al.* [41], which is based on standard statistical means. Prior to both approaches, the experimental dataset of each session was divided into uniform cycles of movement.

According to Schwartz *et al.* [41], the overall reproducibility of a system can be divided into intrinsic and extrinsic errors. The intrinsic error corresponds to the error that occurs naturally in any experiment, *i.e.*, intra/inter-subject variability, and is unavoidable. Conversely, the extrinsic error corresponds to experimental errors, for instance caused by the operator, and which can be subject to improvements. In this study, intra-session variability was considered as the intrinsic error. It was calculated as the mean standard deviation of a cycle in relation to the mean cycle of its own session. Inter-session variability, a source of intra- and inter-operator variability, was considered as the extrinsic error. It was calculated as the mean standard deviation of a cycle in relation to the mean cycle of the five sessions combined. Based on these two (intrinsic and extrinsic) errors, two indexes were extracted. The first index, here called *m*, corresponds to the mean value of the extrinsic error. The second index, here called *r*, corresponds to the ratio of the mean of the extrinsic error (*m*) over the mean of the intrinsic error. In addition, the CMCwp index was used to quantify the precision of the calibrations and the REF method, independently, by taking into account differences in shape, offset, correlation, and range of motion. Since this index behaves in the same way as CMCip, the same quality thresholds are used here. As for the *m* index, the CMCwp was calculated in order to reflect inter-session variability. The CMCwp index was processed for each subject and then averaged over the 10 subjects. All the precision indexes (CMCwp, *m*, and *r*) were calculated for each MIMU calibration and for the REF, for each movement performed and each joint angle of interest.

## 3. Results

### 3.1. Wrist

For the wrist, the FE and AA angles were studied during the wriFE and wriAA movements respectively. The results on the wheel movement were not processed since this movement mainly relied on elbow flexion/extension and shoulder degrees of freedom (DoFs).

#### 3.1.1. Trueness

**Figure 5:**
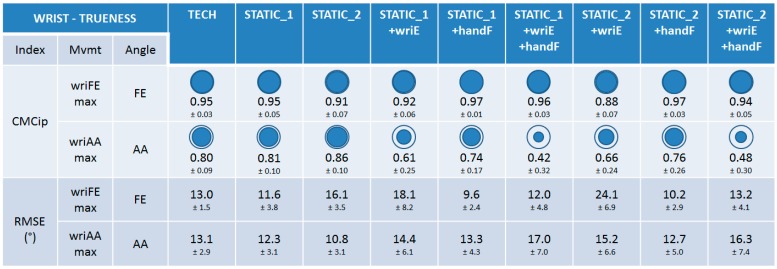
The results on trueness for the wrist joint. CMCip and RMSE values are reported for the different calibrations during maximal wrist flexion-extension (wriFEmax) and abduction-adduction (wriAAmax). For visual clarity, the CMCip mean value is completed with a proportional blue disc included in a blue circle that represents the optimal value 1.

The trueness of the nine calibrations was very good and equivalent (0.88 ≤ CMCip ≤ 0.97) for the wrist FE angle during wriFEmax movement (Figure 5). Lower trueness and disparities between calibrations could be observed for the wrist AA angle during wriAAmax movement. Calibrations that were not composed of the wriE calibration step had good trueness (0.74 ≤ CMCip ≤ 0.86), whereas those composed of it had a bad trueness (0.42 ≤ CMCip ≤ 0.66). Regarding RMSE values, very small discrepancies could be observed between the calibrations for the two movements performed (9.6° ≤ RMSE ≤ 24.1°).

#### 3.1.2. Precision

**Figure 6:**
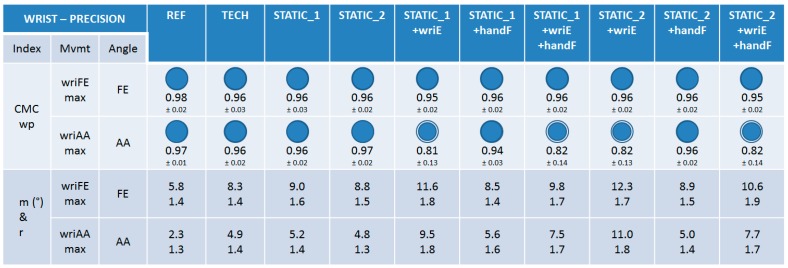
The results on precision for the wrist joint. CMCwp, *m*, and *r* values are reported for the different calibrations and for the REF method during maximal wrist flexion-extension (wriFEmax) and abduction-adduction (wriAAmax). For visual clarity, the mean value of the CMCwp is completed with a proportional blue disc included in a blue circle that represents the optimal value 1.

The precision of the nine calibrations was very good and equivalent (to each other and to the REF), considering mean CMCwp values (0.94 ≤ CMCwp ≤ 0.97) for the FE and AA angles during wriFEmax and wriAAmax movements, respectively (Figure 6), except for calibrations composed of the wriE calibration step that had lower, but still good, values for the AA angle during wriAAmax (0.81 ≤ CMCwp ≤ 0.82). A similar observation can be made when looking at the m and r indexes. Index m was in the range [8.3°–12.3°] and [4.9°–11.0°] for all the calibrations for wriFEmax and wriAAmax, respectively, with higher values for the calibrations composed of the wriE calibration step in both cases. Index r is reported in the same way and all its values remained relatively low (1.3 ≤ r ≤ 1.9). Note that the REF method was not subject to any operator variability, thus the precision results must be interpreted with this information in mind.

### 3.2. Elbow

For the elbow, the FE angles were studied during the elbFE and wheel movements and the PS angles during the elbPSmax movement.

#### 3.2.1. Trueness

**Figure 7:**
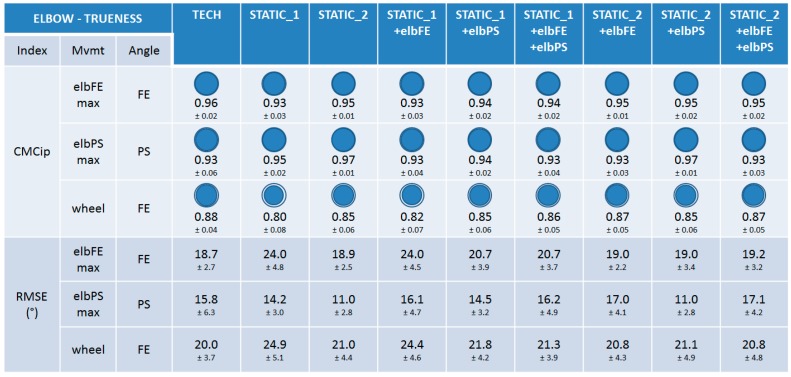
The results on trueness for the elbow joint. CMCip and RMSE values are reported for the different calibrations during the maximal elbow flexion-extension (elbFEmax), maximal forearm pronation-supination (elbPSmax), and wheel movements. For visual clarity, the CMCip value is completed with a proportional blue disc included in a blue circle that represents the optimal value 1.

When examining the CMCip index it can be seen that the trueness of the nine calibrations was very good (0.93 ≤ CMCip ≤ 0.97) for the elbow FE angle during the elbFEmax movement and the forearm PS angle during elbPSmax (Figure 7). Slightly lower, but still good, CMCip values were reported for the elbow FE angle during the wheel movement (0.80 ≤ CMCip ≤ 0.88). No particular discrepancy could be observed between the calibrations for any of the three movements performed, when examining both the CMCip and RMSE indexes (11.0° ≤ RMSE ≤ 24.9°).

#### 3.2.2. Precision

**Figure 8:**
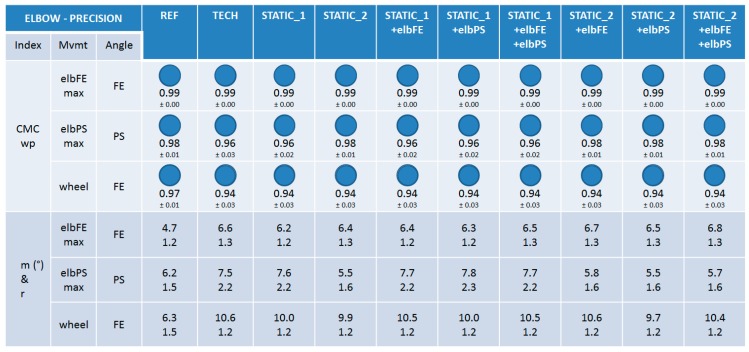
The results on precision for the elbow joint. CMCwp, *m*, and *r* values are reported for the different calibrations and the REF method during the maximal elbow flexion-extension (elbFEmax), maximal forearm pronation-supination (elbPSmax), and wheel movements. For visual clarity, the CMCwp value is completed with a proportional blue disc included in a blue circle that represents the optimal value 1.

When examining the CMCwp index, the precision of the nine calibrations was very good for the elbow FE angle during the elbFEmax and wheel movements and for the forearm PS angle during the elbPSmax (0.94 ≤ CMCip ≤ 0.99) (Figure 8). When examining the m and r indexes, very good precision could also be observed for all the calibrations, with indexes m and r in the ranges [5.5°–10.6°] and [1.2–2.3], respectively, for all the angles inspected. No particular difference could be observed between the calibrations when examining all the indexes, except m and r values were better for calibrations composed of STATIC_2 for PS angles during elbPSmax (Figure 8).

### 3.3. Shoulder

For the shoulder, the FE, AA, and IER angles were studied during the shoF, shoA, and wheel movements. CMCip values were not judged interpretable for the AA angle during the shoFmax and shoAmax movements due to the limited range of motion present for this angle.

#### 3.3.1. Trueness

**Figure 9:**
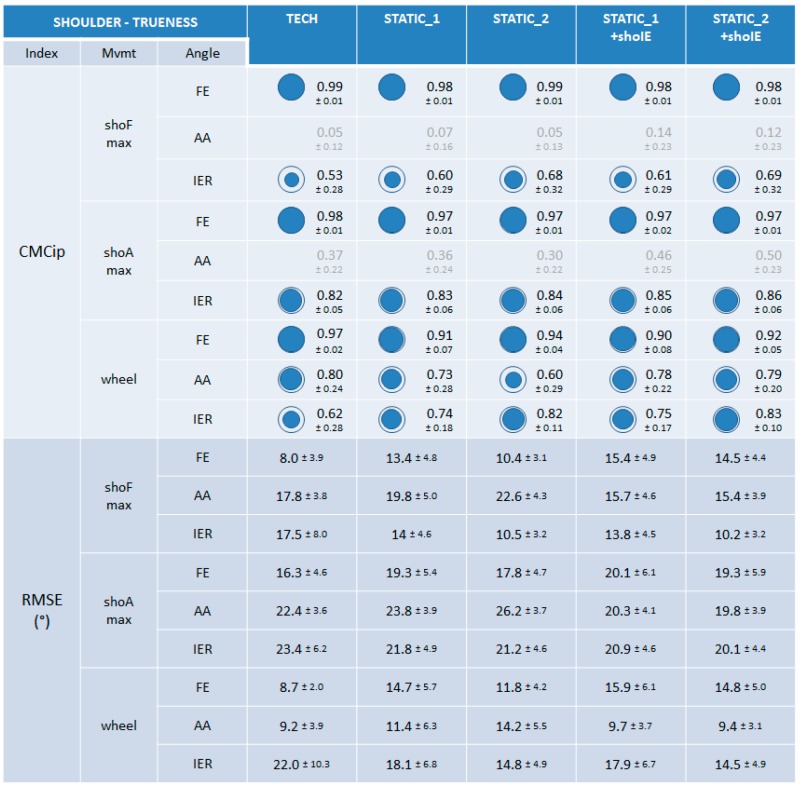
The results on trueness for the shoulder joint. CMCip and RMSE values are reported for the different calibrations during the maximal shoulder flexion (shoFmax), maximal shoulder abduction in the scapular plane (shoAmax), and wheel movements. For visual clarity, the CMCip value is completed with a proportional blue disc included in a blue circle that represents the optimal value 1.

When examining the CMCip index, the trueness of the five calibrations was very good for the shoulder FE angle throughout the three movements performed (0.90 ≤ CMCip ≤ 0.99) (Figure 9). It should be noted that FE was interpreted as the principal movement during shoAmax due to the Euler sequence used (*ZXY*). Lower trueness and disparities between the calibrations could be observed for the AA and IER shoulder angles during shoFmax, shoAmax, and wheel movements (0.53 ≤ CMCip ≤ 0.86). No constant predominance of a particular calibration could be noticed. Nevertheless, the lower CMCip values were obtained for TECH for the IER angle during shoFmax and wheel movements. Regarding RMSE values, no particular difference could be observed between calibrations for any of the movements performed (8.0° ≤ RMSE ≤ 26.2°).

#### 3.3.2. Precision

**Figure 10:**
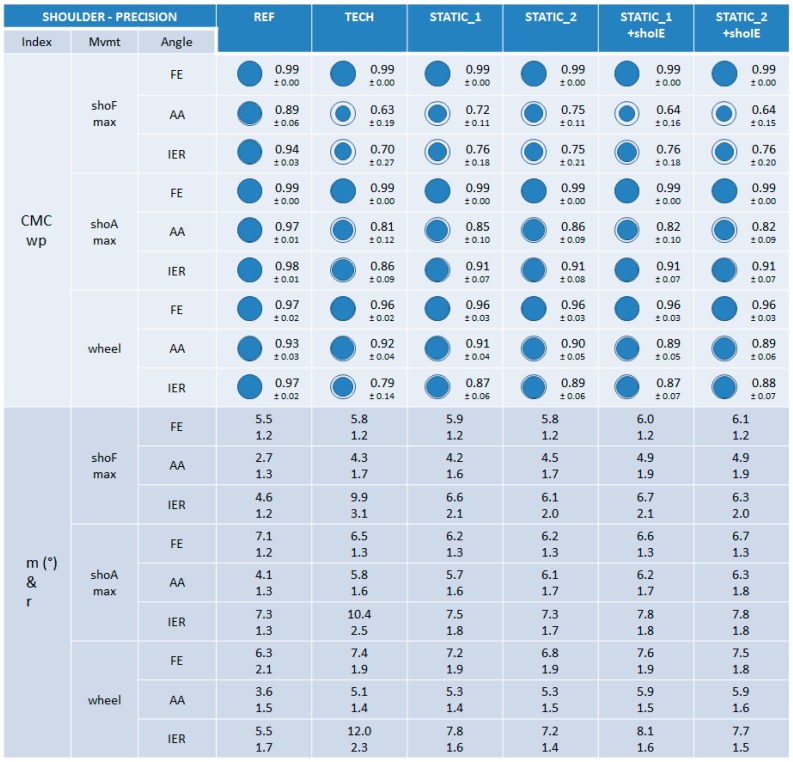
The results on precision for the shoulder joint. CMCwp, *m*, and *r* values are reported for the different calibrations and for the REF method during maximal shoulder flexion (shoFmax), maximal shoulder abduction in the scapular plane (shoAmax), and wheel movements. For visual clarity, the CMCwp value is completed with a proportional blue disc included in a blue circle that represents the optimal value 1.

When examining the CMCwp index, the precision of the five calibrations was very good and equivalent for the shoulder FE angle throughout all the three movements performed (0.96 ≤ CMCwp ≤ 0.99) (Figure 10). Generally lower precision for the AA and IER angles for all the calibrations could also be observed (0.63 ≤ CMCwp ≤ 0.92), with systematically poorer values for the TECH calibration for the IER angle.

Observation of the m and r indexes confirms the results from CMCwp, meaning the precision of calibrations was globally very good and equivalent ([4.2°–8.1°] and [1.2–2.1] respectively), except for the TECH calibration for the IER angle for all the three movements performed ([9.9°–12.0°] and [2.3–3.1], respectively) (Figure 10).

## 4. Discussion

### 4.1. Goal and General Results

The aim of this study was to compare sensor-to-segment calibrations for the MIMU-based estimation of wrist, elbow, and shoulder (humero-thoracic) joint angles. Calibrations were selected from the literature, mainly from the work of De Vries *et al.* [9], and included TECH, STATIC and FUNCT classes of calibration. For each joint, the comparison was made by examining both trueness and precision validity criteria.

This study demonstrated that only very small discrepancies occurred between calibrations, with the exception of a few cases, and that MIMU-based upper limb joint angle estimation can be performed with good trueness and very good precision.

It should be mentioned that the error values presented in this study are specific to this experiment. The whole assessment of MIMU-based upper limb kinematic data relies on the Xsens data fusion algorithm. Although other fusion algorithms or alternative proposals exist in the literature [11,14,21], the results presented in this study reveal a trend in performance evaluation in terms of trueness and precision for experiments performed under similar conditions. Concerning the comparison of calibrations, the comparison between them remains valid as long as the same procedure was used.

### 4.2. Differences between Calibrations

Concerning the wrist joint, lower trueness and precision for the calibrations composed of the wriE calibration step (wrist extension on a table) may be explained by the difficulty for subjects of performing this movement “purely” (*i.e.*, in the “up/down” direction). Based on feedback from the operators and subjects, this movement was often composed of a small unexpected abduction/adduction movement and appeared difficult to reproduce. This result is inconsistent with De Vries’ work which showed low dispersion of the segment axis estimated during this calibration step (2.9° ± 0.5) [9]. The use of a vertical slab to guide the vertical trajectory of this movement would probably increase its reproducibility. Concerning the elbow joint, the only difference observed between calibrations concerned better precision for calibrations composed of the static STATIC_2 during elbPSmax movement. Indeed, this movement was performed in the STATIC_2 pose (*i.e.*, with an elbow flexion of 90°). Thus a less unpredictable STA effect (most certainly acting on the MIMU sensor placed on the upper arm) was possibly involved in the joint angle estimation based on a calibration composed of STATIC_2 than for the others. Concerning the shoulder joint, disparities in the trueness results between calibrations could be observed for the coupled AA and IER shoulder angles throughout the three shoulder movements performed (shoFmax, shoAmax and wheel), without any constant predominance of a particular calibration. Concerning precision, TECH calibration appeared less precise than the others when examining the coupled IER angle for all the shoulder movements performed. Further analysis performed for the elbow joint during the wheel movement highlighted the same observation, leading to the reasoning that the MIMU sensor placed on the upper arm might be the cause of lower precision around the longitudinal axis (IER).

Generally, only small differences between calibrations could be observed. Specifically, the STATIC and FUNCT classes of calibration demonstrated similar precision results, as had already been shown on the lower limb using the optoelectronic system [42]. It should be mentioned that the MIMU-based joint angles estimated using all the calibrations tested were all dependent on the MIMU sensor placement and attachment performed by the operator. Thus they were all subject to the same STA effects. In addition, all the calibrations were made with the same assumption concerning the orientation of the MIMU sensor placed on the thorax, *i.e.*, that the MIMU sensor was “perfectly looking forward” (*i.e.*, its technical *Z* axis was in the sagittal plane). The more rigorous and the less sensitive to STA the placement and attachment of the MIMU sensors are, the better the quality of the final joint angle estimation.

### 4.3. Specificities for TECH Calibration

TECH calibration presented globally good trueness and precision results for all the joints studied, except for the shoulder. These good results were directly related to the capacity of the operator to place the MIMU sensors in the proper (in the anatomical meaning) orientation on the segments and reproduce it. Indeed, placing MIMU sensors in order to ensure that the sensor edges are parallel to the segment can be done without great difficulty on the hand, forearm segments, and thorax segments. The placement on the upper arm segment is more difficult, although written instructions were given to the operator (medio-laterally, with the MIMU sensor length oriented in the direction of the longitudinal axis).

### 4.4. Comparison with the Literature and Limits of the Study

Concerning trueness (“close to the reference”), the results highlighted that most of the sensor-to-segment calibrations can involve joint angles similar to those obtained by the REF method. The CMCip index allowed quantifying the similarity between each MIMU-based joint angle curve and the REF joint angle curve, by taking into account differences in shape, offset, correlation, and range of motion. Although the RMSE index is straightforward to interpret (in degrees), it mainly informs on a difference of offset and must be considered as a secondary index. It should be mentioned that differences between MIMU-based and REF joint angle always exist due to the different definitions of the segment axes and the dissimilar effect of STA [9]. All the RMSE values reported in this study are in the range of [8.0°–26.2°] and comparable to previous results of 20° and 10°–20° reported in the literature [9,11]. Referring MIMU-based data to optoelectronic system-based ones (REF) conforming to ISB standards is certainly of interest; however, it is important to keep in mind that this reference can be called into question for several reasons. First, all non-invasive techniques suffer from STA effects, so reference data is not systematically representative of the underlying bone movements [27]. Second, the kinematics computations associated with the REF necessarily rely on arbitrary choices. Indeed, several scientific points are still open to discussion regarding ISB standards, such as the estimation of the gleno-humeral joint center and the definition of the hand segment CS. Third, other measurement protocols associated with the optoelectronic system have been proposed in the literature, such as the CAST method [6] and functional approaches [25]. These two examples of alternatives are judged less sensitive to STA and more representative of real joint motion by these authors, respectively. Any change in the REF method would certainly affect the final reference joint angles, and so the final trueness results of MIMU-based joint angles. If trueness is the principal objective of a study, the proposal of Picerno *et al.* [13], which associates a segment definition based on palpable anatomical landmarks with MIMU sensors, represents an interesting alternative.

On the contrary, assessing the precision of joint angles (reproducibility) is not dependent on the reference and is therefore less subject to discussion. It should be emphasized that no confidence can be given to a non-reproducible system and therefore precision should be considered a key criterion when evaluating the performance of MIMU-based systems.

Precision scores for the REF method were necessarily higher than for the MIMU-based joint angle estimation since operator variability was not considered (markers remained constant for all the sessions). The values of CMCwp, *m*, and *r* indexes for the REF method were slightly higher than those presented by Vanezis *et al.* [43], especially for the wrist joint angles. These higher values can be explained by the higher range of motion (RoM) of the movements performed. Indeed, such statistical indexes, especially CMCip and CMCwp, should be used with great caution for signals with a very low RoM (e.g., wrist movements during wheel) and should not be dissociated from the RoM factor [43]. 

Concerning the MIMU-based precision, the CMCwp, *m* and *r* indexes all presented very good precision in most of the cases studied. Most of the CMCwp values were higher than 0.85. Index *m* quantified the general extrinsic error of this experiment in degrees, by taking account of inter-session variability. The intra- and inter-operator variabilities might have been assessed separately but this was not the objective of the present study. Index *r* informed on the possibility to reduce the extrinsic error (inter-session), by comparing it to the unavoidable intrinsic error (intra-session). The lower index *r* is the closer index *m* is to the minimal error and thus the better the precision of the procedure. The results showed that the global precision of MIMU-based upper limb joint angles was in the range 5°–10°, under extended human variability conditions (subject and operator). Most of the values of index *r* were lower than 2.0, highlighting that the procedures had a high level of precision [43], except for the TECH calibration for the shoulder joint. It cannot be excluded that the fixed operator order A-B-A-C-A might play a role in the general low inter-session variability. However, it should be underlined that all the subjects were asked to strictly follow the operator’s instructions and not to communicate any information from the previous sessions. All the calibration sessions were interspaced with each other by a test session (lasting at least 30 min). The use of an elastic band on the subject’s skin prevented any hints of MIMU sensor placement from one operator to the next. Finally, according to the subject’s feedback, no influence from one operator to another was present. In the authors’ opinion, the most consistent explanation for these very good precision results was the common knowledge and instructions communicated to the operators. First, five calibration pre-sessions were performed on two different subjects (not included in this study) by the three operators during the month before this study. Second, each operator had dedicated written instructions during the calibration session. Adding a novice operator to this study would have ensured that the high precision reported could be attributed (or not) to the rigorousness of the experimental protocol. Parel *et al.* [28] suggested three training subjects to obtain confidence in their experimental protocol using ISEO, which is a similar MIMU-based protocol for upper limb kinematics. We make similar recommendations with three pre-sessions performed on different subjects as well as the use of written instructions during the experiment.

## 5. Conclusions and Outlook

This study compared sensor-to-segment calibrations for the MIMU-based estimation of wrist, elbow, and shoulder (humero-thoracic) joint angles. No particular discrepancy between calibration classes (TECH, STATIC, FUNCT) was highlighted, except poorer results for TECH for the shoulder. Moreover, this study characterized the global accuracy of MIMU-based upper limb joint angles (trueness and precision) under extended human variability conditions (subject and operator), demonstrating a good trueness (“close to the reference”) and a very good precision (reproducibility) in the range of 5°–10°. The overall accuracy of MIMU-based joint angle data seems to be more dependent on the level of rigor of the experimental procedure (operator training, STA awareness, *etc.*) than on the choice of calibration itself. Other validity criteria that take into account the final interpretation of joint angle data, such as a limited cross-talk effect [8,44] and amplitude coherence [37], could provide more keys to researchers in their final choice of calibration. 
